# Boron doped graphene wrapped silver nanowires as an efficient electrocatalyst for molecular oxygen reduction

**DOI:** 10.1038/srep37731

**Published:** 2016-12-12

**Authors:** Anju K. Nair, Vineesh Thazhe veettil, Nandakumar Kalarikkal, Sabu Thomas, M. S. Kala, Veena Sahajwalla, Rakesh K. Joshi, Subbiah Alwarappan

**Affiliations:** 1 International and Inter University Centre for Nanoscience and Nanotechnology, Mahatma Gandhi University, Kottayam, 686 560, Kerala, India; 2 Department of Physics, St Teresas’s College Ernakulam, 682011, Kerala, India; 3 CSIR- Central Electrochemical Research Institute (CSIR-CECRI), Karaikudi, 630 006, Tamilnadu, India; 4 School of Pure and Applied Physics, Mahatma Gandhi University, Kottayam, 686 560, Kerala, India; 5 School of Chemical Sciences, Mahatma Gandhi University, Kottayam, 686 560, Kerala, India; 6 Centre for Sustainable Materials Research and Technology (SMaRT), School of Materials Science and Engineering, University of New South Wales Sydney, NSW, Australia

## Abstract

Metal nanowires exhibit unusually high catalytic activity towards oxygen reduction reaction (ORR) due to their inherent electronic structures. However, controllable synthesis of stable nanowires still remains as a daunting challenge. Herein, we report the *in situ* synthesis of silver nanowires (AgNWs) over boron doped graphene sheets (BG) and demonstrated its efficient electrocatalytic activity towards ORR for the first time. The electrocatalytic ORR efficacy of BG-AgNW is studied using various voltammetric techniques. The BG wrapped AgNWs shows excellent ORR activity, with very high onset potential and current density and it followed four electron transfer mechanism with high methanol tolerance and stability towards ORR. The results are comparable to the commercially available 20% Pt/C in terms of performance.

Scarcity of fossil fuel to meet the ever increasing global energy crisis had made the researchers to think about safe, green and alternate sustainable energy resources that will fulfil the need^1^. Fuel cells are highly efficient and sustainable electrochemical energy conversion devices with broad applications in both electronic and portable electronics, capable of generating electricity directly from chemical energy without combustion^2^,^3^. The oxygen reduction reaction (ORR) is a key process in fuel cells, but the sluggish reaction kinetics of the cathodic oxygen reduction reaction considerably limits the efficiency and performance of electrochemical energy conversion^2^. Till date, Pt based materials are considered to be the highly active and effective catalyst for ORR. However, their higher cost, scarcity and detrimental environmental effects have restricted the large scale application of fuel cells^4^,^5^. So, it is the need of the hour to develop a low-cost, safe and stable electrocatalytic materials as an efficient alternatives for the Pt-based fuel cell catalysts^6^,^7^. Recently, tremendous scientific efforts have been put forth to develop electrocatalysts devoid of Pt for fuel cell applications. Various metal nanoparticles such as Au, Ag, Pd and Ni have been explored in detail to attain high ORR activity compared to Pt based catalysts^8^,^9^. Of these materials, Ag nanoparticles with different morphologies have attracted much attention. Ag nanoparticles are widely explored due to their excellent electrochemical activity, high natural abundance and high extinction coefficients, comparatively cheaper than Pt or Au. Moreover, Ag nanoparticles are electrodynamically stable at high pH and extensive durability towards ORR under alkaline conditions. As a result of all these favourable attributes, Ag is considered as an excellent candidate than Pt based electrocatalysts^10^,^11^. The catalytic activity and stability of metal nanoparticles depends on the shape, size, and surface composition of the nanoparticles^12^. Moreover, Ag nanoparticles with different morphology follow the ideal four-electron reduction pathways during ORR^13^,^14^. Amongst various available geometries, one dimensional Ag nanowires exhibits excellent electrical conductivity, thermal stability and structure dependent optical properties and are promising candidates for fuel cell applications^15^. In a recent report, AgNWs with small diameter exhibited excellent ORR activity in hydroxide exchange membrane fuel cells^16^. In another work, researchers evidenced that the high aspect ratio silver nanowires is one of the reason for their superior oxygen electro reduction under alkaline conditions than those of low aspect ratios^17^. Carbon supported silver nanowires cathodes show better catalytic performance than Ag/C nanoparticles in direct borohydride fuel cells^18^.

On the other hand, bare metal nanoparticles have been found to easily aggregate due to their strong vander Waals force between them, which decrease their surface energy and thereby minimizes its catalytic activity. Therefore, suitable supporting substrates are essential to overcome these obstacles^19^. Moreover, the supporting substrates offer synergistic effects to the intrinsic properties of the metal nanoparticles and thereby making the hybrids much more attractive in applications than the nanoparticles by itself 20. Of various substrates available, graphene-a one atom thick two dimensional sheet of sp^2^ hybridized carbon have been widely employed in electrocatalysis due to their excellent electronic conductivity, enormous large surface area, high mechanical strength, excellent carrier mobility and stability^21^,^22^. However, the homogeneous loading of metal nanoparticles on graphene surface is difficult due to its defect free nature and smooth surface, which deteriorates the catalytic activity of nanoparticles. Recent studies confirmed that the doping of heteroatoms such as nitrogen, boron, sulphur or phosphorous in to graphene sheets have shown superior electrocatalytic activities due to the doping induced charge polarization^23^,^24^. Both experimental and theoretical calculations have confirmed that the doping provides abundant active sites that facilitate changes in the local charge density and spin density re-distribution of the carbon network which in turn enhances the ORR activity^25^,^26^. Furthermore, heteroatom doping has also been found to enhance the interaction between metal nanoparticles and doped graphene support to further improve the electrocatalytic activity and stability of the hybrids upon comparison with undoped counterparts^27^,^28^.

Of various surface variant methods available, boron doping is extensively studied^29^,^30^. The introduction of boron increases the number of hole-type charge carriers. In addition, the incorporation also activates the two dimensional carbon materials by conjugating the carbon π electrons with electron-deficient boron^31^. Recent investigations have suggested that boron doped graphene (BG) effectively promotes ORR as a result of lower electronegative character of boron than carbon^30^,^32^. Furthermore, the positively polarized boron atoms attract the negatively polarized oxygen atoms, leading to chemisorption^33^. Ferrighi *et al*. had investigated boron doping by DFT calculations. Their results confirmed that the local high spin density on the basal plane was enhanced as a result of boron doping. Further, the incorporation of boron facilitates the adsorption of oxygen and -OOH molecules and enhances the ORR activity^34^. The B-doped carbon nanotubes also exhibited enhanced oxygen reduction performance due to the interaction of π electrons in the conjugated carbon system arising from boron doping along with improved O_2_ adsorption^35^,^36^. Recently, our group demonstrated a novel route for the synthesis of boron doped graphene from boron carbide (B_4_C) and is found to be an effective bi-functional catalyst for ORR and OER applications^37^. Till date, only a very few studies have focussed on the growth of metal nanoparticles over boron doped graphene. Recently, Rao *et al*. had reported that TiO_2_ nanoparticles with B-doped graphene can alter the effective band gap of the composites and thereby enhances the photocatalytic efficiency^38^. In another work, Cheng *et al*. evidenced the oxygen reduction reaction of Ag nanoparticles at various proportions supported on boron doped multi-walled carbon nanotubes^39^. Further, Yongrong *et al*. evidenced that the activity of uniformly loaded Pt nanoparticles over BG towards methanol oxidation reaction is higher than that of Pt/G nanoparticles and Pt/C nanoparticles^40^.

Herein, we developed a facile two step process to prepare boron doped graphene sheets (BG) that support the growth of AgNWs (denoted as BG-AgNW). AgNWs decorated boron doped graphene have been successfully synthesized through an *in situ* route. The strong adsorption and the partial reduction of boron doped graphene sheets towards metal ions in the solution offer the initial nucleation sites. Furthermore, it also enhances the growth of long metal nanowires and thereby it facilitates the charge transfer^41^. Moreover, this one step process resulted in the formation of interconnected graphene-AgNWs networks without any interface issues^42^. To the best of our knowledge, till date there are no reports on the *in situ* reduction of AgNWs over boron doped graphene sheets for ORR applications. Furthermore, the integration of Ag nanowires onto BG sheets resulted in a very high electrocatalytic activity and stability towards ORR and it follows a four-electron pathway with a very low yield of peroxide.

## Results and Discussion

**Figure 1:**
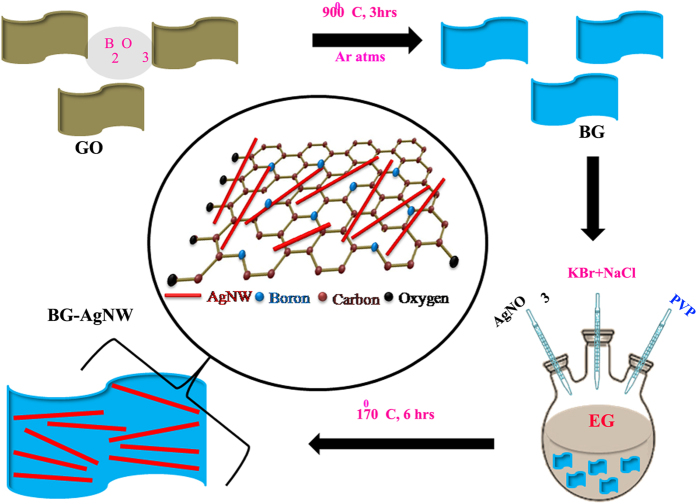
Schematic illustration of the synthesis procedure of BG-wrapped AgNWs.

The methodology followed in this work to develop BG-AgNW hybrid includes two steps: (i) doping of boron in to the graphene sheets under inert atmosphere (ii) *in situ* synthesis of AgNWs over BG sheets. BG was prepared by the thermal-annealing of graphene oxide in the presence of B_2_O_3_ at 900 °C for 3 h in Ar atmosphere. After annealing, atomic rearrangement occur together with the incorporation of boron atoms in to the graphene matrix. This resulted in the growth of Ag nanowires on BG by polyol mediated synthesis that employ AgNO_3_ as the metal precursor. The polyol synthesis is based on the reduction of an inorganic salt by a polyol at an elevated temperature. Herein, a surfactant is used to avoid agglomeration of the nanoparticles. Polyol process allows control over the diameter and length of Ag nanowires^43^. For comparison, a reduced graphene oxide silver nanowire (RG-AgNW) hybrid is also prepared by the similar procedure but without adding boron oxide. Figure 1 illustrates the procedure scheme for synthesizing silver nanowire decorated boron doped graphene sheets. The experimental procedure is detailed in the Experimental Section.

The *in situ* growth of AgNWs over boron doped graphene sheets was observed by UV-Vis absorption spectroscopy and is shown in Supplementary Fig. S1. GO shows a strong absorption peak at 230 nm due to the π-π^*^ transition of the C-C aromatic rings and a shoulder at 300 nm owing to the n-π^*^ transition of the C=O bond^44^,^45^. After the reduction reaction, the absorption spectrum of BG shows a maximum absorption at 247 nm and the shoulder at 300 nm disappeared, indicating the reduction of GO. The red shift noticed herein is due to the increased electron density witnessed due to the removal of sp^3^ hybridised carbon. Further, this observation is consistent with the restoration of sp^2^ hybridised carbon atoms^44^. This transfer indicated that the electronic conjugation in the graphene sheets was restored. The BG-AgNWs exhibit strong absorption peaks at 247 nm, 350 nm and 386 nm confirming the successful formation of BG-AgNWs hybrid. The peak at 350 nm and 386 nm can be attributed to the surface plasmon resonance peaks of the long AgNWs. Moreover, the strong absorption peak at 386 nm is due to the transverse plasmon effect associated with AgNWs^46^.

**Figure 2:**
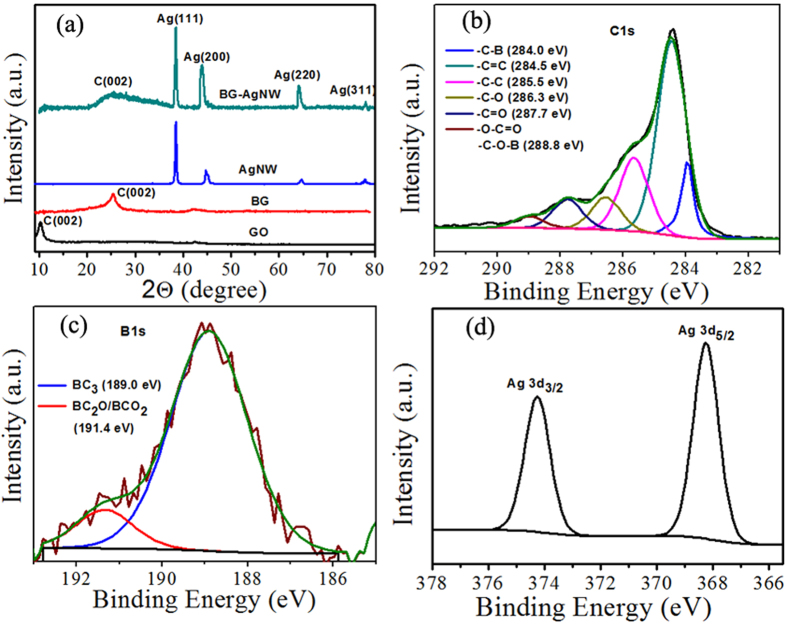
(**a**) XRD patterns of GO, BG, AgNWs and BG wrapped AgNWs (**b**) C1s spectrum (**c**) B 1 s spectrum and (**d**) Ag 3d spectrum of BG wrapped AgNWs.

In order to examine the crystallinity of the samples, powder X-ray diffraction (XRD) technique was carried out. Figure 2a depicts the XRD pattern of GO, BG and BG-AgNW. GO exhibits a strong peak at 10.23° corresponding to an interlayer spacing of 0.86 nm, while BG displayed a characteristic (002) peak at around 25.47° with a d-spacing of 0.34 nm indicating the effective de-oxidation of GO during BG synthesis thereby confirming its high crystallinity. The characteristic diffraction peaks of AgNW, BG-AgNW and RG-AgNW (see Supplementary Fig. S2) observed at 38.4°, 44.1°, 64.2°, 77.8° could be indexed to (111), (200), (220) and (311) planes of the face centred cubic structure of AgNWs (ICDD No: 04-0783)^47^. The Ag (200) and Ag (222) XRD peaks of BG-AgNW hybrids exhibited a sharper peak, while the bare AgNW displayed broader Ag (200) and Ag (222) peaks. The *in-situ* formation of AgNW over the BG surface enhanced the crystallinity and exhibits sharper Ag peaks. Moreover, BG nanosheets have high surface energy and strong interaction between AgNWs precursors which might have caused rapid heterogeneous nucleation and thus exhibits large crystallinity^48^. Upon comparison with BG, the C (002) peak position of BG-AgNW gets broader and shifts to 24.98°, suggesting the successful insertion of AgNWs. This insertion prevents the restacking of BG. The peak broadening and the increased d-spacing of C (002) peak can also be attributed to the more interaction of AgNWs with the BG sheets, which is in excellent agreement with the literature reports^49^,^50^. For example, Jiang *et al*. reported that the XRD spectrum of silver nanoparticles incorporated nitrogen doped graphene exhibit broad reflection at ∼26° corresponds to C (002) peak^49^. Zhao *et al*. evidenced that the XRD pattern of Ag–RGO consists of a broad reflection C (002) plane at 24.21° along with the fcc crystal planes of Ag nanoparticles^50^. Moreover, the growth of AgNWs plays a key role as a spacer to avoid the re-stacking of the individual graphene sheets^51^. From the above results, it is evident that the formation of AgNWs on boron doped graphene sheets is successful.

In order to investigate the surface chemical state of the hybrid material, X-ray photoelectron spectroscopy (XPS) analysis was carried out. Figure S3 in the supplementary information shows the wide scan survey spectra of the BG-AgNWs hybrid, which confirmed the presence of C, O, B and Ag elements. Figure 2b depicts the corresponding deconvoluted C1s peak of BG-AgNW hybrids. The most dominant peak observed at 284.5 eV (C=C) is the signature of the sp^2^ bonding whereas the small peak located at 285. 5 eV (C-C) corresponds to sp^3^ carbon due to the grain boundaries and defects in the lattice structure^52^. The C1s spectrum also shows a peak located at 284.1 eV, which is assigned to C-B bond indicating the formation of boron– carbon bonds in the BG lattice^40^. The other peaks located at 286.3 eV, 287.7 eV, and 288.8 eV can be attributed to the hydroxyl groups and boron oxycarbides respectively^37^. The chemical state of the doped boron in BG-AgNW has also been explored using deconvoluted B1s spectra shown in Fig. 2c. The atomic percentage of BG in BG-AgNW is calcuated to be 1.5%. The presence of B in BG is evident from the binding energy peak at 189.0 eV assigned to BC_3_ and it confirms that boron predominantly exists as BC_3_^53^. The other peak located at 191.4 eV is due to boron oxy carbides. Figure 2d shows the Ag 3d spectrum, and the peaks shown in 368.2 eV (Ag 3d 5/2) and 374.2 eV (Ag 3d 3/2) provide direct evidence for the formation and decoration of AgNWs over BG sheets.

**Figure 3:**
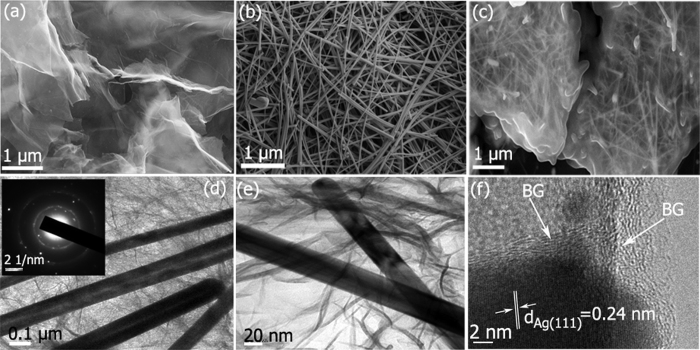
FESEM images of (**a**) BG (**b**) AgNWs (**c**) BG wrapped AgNWs. TEM images of (**d**) Low resolution image of BG wrapped AgNWs (the inset shows the corresponding SAED pattern) (**e**) High resolution image of BG wrapped AgNWs (**f**) HRTEM image of BG wrapped AgNWs.

The structure and morphology of the as prepared BG wrapped AgNWs were investigated by field emission scanning electron microscope (FESEM) and transmission electron microscope (TEM). The FESEM image of B-doped graphene after thermal annealing showed a wrinkled and rippled surface structure (Fig. 3a). The FESEM of BG wrapped AgNWs image clearly indicates that the ultra long thin AgNWs are homogeneously embedded inside the BG sheets (Fig. 3c). It is evident that the AgNWs were completely wrapped by BG sheets. However, the embedded AgNWs retained its geometry (wire like structure) with an average diameter of 60 ± 5 nm and a length up to 10 ± 3 μm on the surface of BG sheets as compared with AgNWs and is shown in Fig. 3b. Moreover, the density of AgNWs formed on the surface of BG is very high and thereby it indicates that the B-doping effectively increased the interaction between the BG and AgNW. In addition, the interaction enhances the dispersion of AgNWs over the BG surface. The extent of elemental doping was confirmed by energy- dispersive x-ray (EDX) analysis. The EDX elemental mapping also confirmed the coexistence of AgNWs on the surface of BG (see Supplementary Fig. S4). The EDX profile of BG-AgNW depicted the presence of C, O, B and Ag elements and silicon (originating from the silicon wafer) (see Supplementary Fig. S5). In addition, on the surface of BG-AgNW, various nano channels were created to enhance the specific surface area and to allow the transport of molecules in order to facilitate the catalytic activity.

Transmission electron micrographs reveal the stacking of exfoliated BG sheets and are shown in Supplementary Fig. S6. The observed transparent and wrinkled flake like morphology is similar to RG (see Supplementary Fig. S7). The high-resolution TEM (HRTEM) image of BG (see Supplementary Fig. S8) depicts well distinct graphitic lattice fringes, with an interlayer distance of ~0.36 nm confirming the crystalline nature of the BG nanosheets. The selected area electron diffraction (SAED) pattern of the BG (inset of Fig. S8) shows a ring-like diffraction pattern, which is the characteristic pattern exhibited by the hexagonal lattice of carbon representing the high crystallinity. Figure 2d is the representative TEM image of the BG wrapped AgNWs which indicates that AgNWs are confined to the surface of 2D boron doped graphene sheets with uniform distribution. The inset in Fig. 3d represents the corresponding selected area electron diffraction pattern of the BG wrapped AgNWs. The characteristic diffraction spots in the SAED pattern are due to the single crystalline nature of AgNWs. The detailed structure of the as- synthesised BG wrapped AgNWs was analysed in detail using HRTEM. Figure 3e confirms that the individual AgNWs has been wrapped by graphene sheets which are consistent with FESEM and HRTEM results. Figure 3f is the HRTEM image of the BG wrapped AgNWs. The well resolved fringes with a lattice spacing of 0.24 nm can be ascribed to the (111) plane of the AgNWs which further confirmed the crystalline nature of AgNWs on BG.

**Figure 4:**
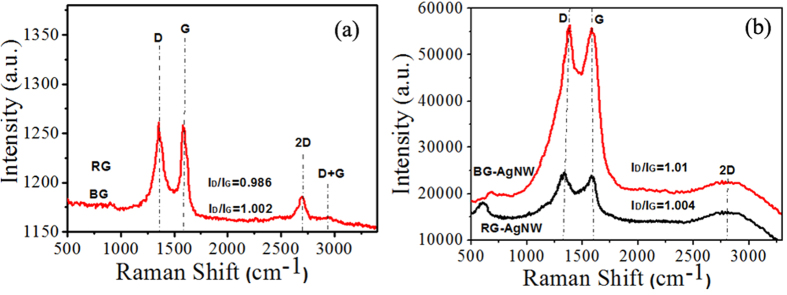
Raman Spectra (excitation wavelength λ = 532 nm) of (**a**) RG and BG (**b**) RG-AgNW and BG-AgNW.

Raman spectroscopy was used to further investigate the structure of BG and the interaction between AgNWs and BG sheets. Raman spectroscopy is the most effective and non-destructive technique to determine the defects and disordered structure of carbon based materials^54^. Herein, the Raman spectra of BG exhibited an intense G peak at 1577.55 cm^−1^ and a wide 2D peak at 2700 cm^−1^ which confirmed the formation of few layered BG with graphitic structure (Fig. 4a). The presence of a highly intense D band at 1353.04 cm^−1^ in BG samples clearly suggested the existence of several defects in the graphene layers. These defects are commonly ascribed to the severe oxidation of graphite and boron doping in the carbon hexagonal lattice. However, in the case of reduced graphene, the G band and 2D band are located at 1579.65 cm^−1^ and 2694.64 cm^−1^ respectively. Further, we witnessed no considerable shifts or line broadening after boron doping and this confirm that the graphene structure is retained even after boron doping. In addition, the larger I_D_/I_G_ value (1.002) for BG compared with RG (I_D_/I_G_ = 0.986) confirmed the presence of more defects in BG than the pristine graphene prepared under similar conditions. Moreover, BG exhibited a broader and up-shifted band around 2700 cm^−1^ thereby confirming the formation of few-layered BG in this work.

Further, as evident from Fig. 4b, the intensity of the D band (1359.32 cm^−1^) and G band (1583.33 cm^−1^) for BG have been drastically enhanced after the inclusion of AgNWs due to the greater Raman scattering cross sections of the high density metallic nano gaps. The presence of D band in the Raman spectra of both RG-AgNW and BG-AgNW indicates that the density of the defects are similar in both the case due to the introduction of vacancies during the insertion of AgNWs. The intensity ratios of the D to G band (ID/IG) in RG-AgNW and BG-AgNW were calculated to be 1.004 and 1.01 respectively. Moreover, the enhancement factor for the BG-AgNW hybrid indicated the development of charge transfer complexes involving chemical interaction between AgNWs and the graphene sheets^55^,^56^. Herein, the boron doping resulted in the creation of defects in the graphene system which further increases the electron-hole scattering and minimizes the 2D band density.

### Electrocatalytic performance towards Oxygen reduction reaction

Tailoring the morphology of Ag nanoparticles is one of the important concern to improve the catalytic activity. Herein, we have first investigated and compared the effects of different geometries of AgNWs towards their ORR performance. Herein, we chose three different geometries of silver nanostructures such as nanosphere, nanocubes and nanowire to study their effect on ORR. The electrochemical performance of various geometries of silver nanostructures such as nanowire (AgNW), nanocube (AgNC) and nanosphere (AgNS) with same loading (as mentioned in the electrochemical experiments) are investigated by cyclic voltammerty (CV) measurements in O_2_ saturated 0.1 M KOH solution are shown in Supplementary Fig. S10. The TEM images of AgNS, AgNC and AgNW are shown in Supplementary Fig. S9. The AgNC and AgNS exhibited similar current density with an ORR reduction peak at −0.262 V and −0.308 V respectively. However, in the case of AgNWs, the current density of reduction peak at −0.24 V is −1.09 mA/cm^2^. Of these three, AgNWs shows better catalytic activity in terms of current density and reduction potential. The difference in CV measurements implies that electrocatalytic activity of silver nanostructures should be shape dependent. Specifically, the Ag (100) surface was found to be the most active site in alkaline media. Herein, the AgNWs exhibited outstanding ORR performance though it maintain mainly the (111) structure, due to the distinctive surface electronic properties of the one dimensional metallic nanostructures^57^. Moreover, the weaker adsorption of OH- on silver (111) facet gives more active sites and thereby it leads to the higher catalytic activity of ORR on AgNWs than that on silver nanocubes^58^. The better electrocatalyst AgNWs, among the other nano structures have been prepared *in situ* over boron doped graphene sheets. Further, we have also studied how the incorporation of BG enhances the electocatalytic activity?

**Figure 5:**
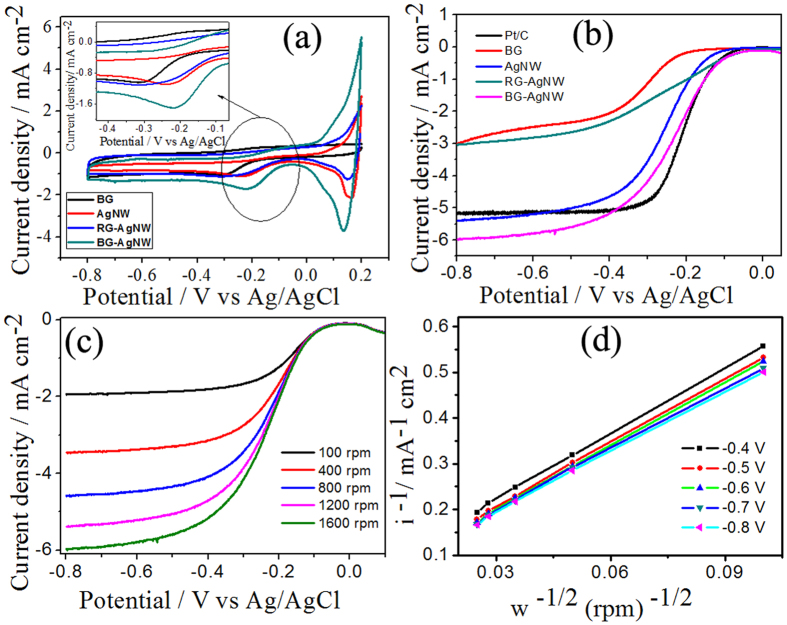
(**a**) CV of BG, AgNW, RG-AgNW and BG-AgNW in O_2_ saturated 0.1 M KOH (the inset displays the zoomed area) (**b**) LSVs of different catalyst in 0.1 M KOH at 1600 rpm (**c**) LSV of BG-AgNWs at different rpm (**d**) K-L plot for BG-AgNWs, Scan rate 10 mV s^−1^.

Electrocatalytic characterization of BG, AgNWs, RG-AgNWs and BG-AgNWs were first investigated by cyclic voltammerty (CV) measurements in O_2_ saturated 0.1 M KOH solution and the representative voltammograms are shown in Fig. 5a. The samples AgNW and BG-AgNWs both showed a pair of redox peaks at an anodic peak potential (Ep_a_) of 0.19 V and a cathodic peak potential (Ep_c_) of 0.085 V which can be attributed to the Ag_2_O/Ag redox couple^17^. After O_2_ purging, the BG wrapped AgNWs showed a well defined characteristic oxygen reduction peak centred at −0.21 V with a current density of −1.76 mA/cm^2^. The peak potentials of oxygen reduction for AgNWs, BG and RG-AgNWs were –0.24 V, −0.3 V and −0.29 V with a current density of −1.09 mA/cm^2^, −0.91 mA/cm^2^ and −1.10 mA/cm^2^. The above cyclic voltammetric studies clearly confirms the enhanced electrochemical activity of BG-AgNWs in terms of both current density and onset potential compared with AgNW, BG and RG-AgNW. The reduction peak potential of BG-AgNW is more positive than that of other metal-graphene ORR catalysts reported in the literature such as Ag/Grn^59^, Ag/GO/C^58^, NG/SNWs^32^, indicating an enhanced ORR process that occurs at BG-AgNW.

In order to assess the ORR kinetics on BG-AgNWs sample, linear sweep voltammetry (LSVs) on a rotating disc electrode (RDE) were measured at different rotating speeds from 100 rpm to 1600 rpm in 0.1 M KOH (saturated with O_2_) and the corresponding voltammograms are shown in Fig. 5c. For comparison, analogous LSV curves for different rpms were obtained for AgNWs, BG, RG-AgNWs and commercial 20 wt% Pt/C (see Supplementary Fig. S11). Remarkably, BG-AgNWs exhibits a very low onset potential, which is comparable with commercial Pt/C and more positive than AgNWs, BG or RG-AgNWs at 1600 rpm (Fig. 5b). The ORR current density of BG-AgNWs is higher than the other samples, which further supports the excellent ORR activity. For example, the ORR current on BG-AgNWs at −0.6 V is −5.88 mA cm^−2^, which is higher than that observed for Pt/C (−5.05 mA cm^−2^), BG (−2.5 mA cm^−2^), AgNWs (−5.15 mA cm^−2^) and RG-AgNWs (−2.8 mA cm^−2^). These results clearly find that the BG-AgNWs shows higher reaction current than the individual counterpart and a lower overpotential value that is very close to that of Pt/C (with an onset potential difference of 10 mV). The undoped RG-AgNWs attribute lower current density and catalytic activity than BG-AgNWs. The introduction of boron doped graphene sheets in to AgNWs significantly improved the catalytic activation due to the increase in conductivity and the availability of abundant surface active sites (in respect to surface area) generated during the ORR process. The porosity and surface area of the samples were examined by Brunauer–Emmett–Teller (BET) measurements. Figure S12 indicates the nitrogen adsorption-desorption pattern of BG, RG-AgNW and BG-AgNW hybrids, along with the corresponding pore-size distribution calculated using the (Barrett-Joyner-Halenda) BJH model (the inset of Fig. S12). The isotherm curves for all the samples exhibited a type IV pattern according to the classification of IUPAC^68^ with H_2_ hysteresis loop in the range of 0.3–0.98 relative pressure. These results confirmed that the hybrid materials possess mesoporous structures. The pore size distribution curves of BG-AgNW and RG-AgNW present pore size in the range from 1 to 10 nm. These mesopores are expected to facilitate the diffusion of reactants in the ORR process. On the other hand, BG exhibited a much broader pore size distribution. The BET surface areas for BG, RG- AgNW and BG- AgNW were found to be 190 m^2^ g^−1^, 220 m^2^ g^−1^ and 270 m^2^ g^−1^ respectively. The higher surface area of BG- AgNW can be attributed to the insertion of AgNWs on both the sides of the BG sheets, thereby decreasing the restacking of the graphene sheets^60^. The observed increase in surface area could be one of the important factors responsible for the enhanced ORR activity. The AgNWs introduced porous channels in the hybrid network and thereby activate the O_2_ gas and OH^−^ ion transport^18^,^43^,^47^. Moreover, the combination of AgNWs and BG provides a synergetic effect for the enhanced ORR activity (BG-AgNW hybrid *vs* BG sheets and AgNWs).

Electrochemical Impedence Spectroscopy (EIS) testing was conducted to evaluate the charge transfer behavior of the BG-AgNW, RG-AgNW, BG and AgNW samples at a potential −0.30 V. The corresponding Nyquist plots are composed of an arc in the high frequency region and exhibit a straight line in the low frequency region (see Supplementary Fig. S13). The frequency of the ac voltage was in the range from 100 KHz to 5 mHz, and the impedance data were fitted to the semicircle for calculating the charge-transfer resistance (R_ct_) values, while the straight line at lower frequencies presented the diffusion behavior of ions in the electrode pores^61^. The measured impedance spectra were fitted on the basis of the equivalent circuit, which is given in the inset of Fig. S13. The calculated R_ct_ values for BG-AgNW, RG-AgNW, AgNW and BG are found to be 92 Ω, 113 Ω, 154 Ω and 180 Ω respectively, this reflects the higher conductivities achieved with the BG-AgNW hybrids. The improved conductivity of BG-AgNW guarantees a fast electron transfer in electrocatalytic reaction process, which is beneficial for ORR.

**Figure 6:**
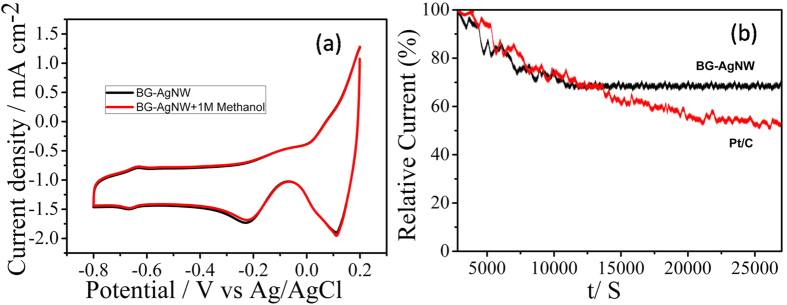
(**a**) Methanol tolerance test for BG-AgNWs and (**b**) stability test for BG-AgNWs catalyst @ −0.4 V.

In order to examine the number of electrons transferred at different overpotentials for BG-AgNWs hybrid, RDE voltammograms at different rotation rates from 100 rpm to 1600 rpm were conducted and the results are shown in Fig. 5c. The kinetic current density in the ORR and electron transfer numbers (n) per O_2_ were determined by the Koutecky-Levich (K-L) equation and the K-L plots (1/i vs 1/ω^1/2^) corresponding to the sample BG-AgNW exhibited a good linearity as shown in Fig. 5d. Further, K–L plots for BG, AgNWs, RG-AgNWs and Pt/C were also compared and shown in Supplementary Fig. S11. For a better understanding, the K–L plots at −0.6 V (@1600 rpm) for different catalysts were shown in Supplementary Fig. S14. From these results, it is evident that BG-AgNWs exhibits a high ORR current, which is higher than that of commercial Pt/C, BG and AgNW. The Tafel plots were derived to further investigate the kinetic differences in ORR catalysis exhibited by BG, RG-AgNW, Pt/c and BG-AgNW (see Supplementary Fig. S15). All the plots depicts two distinct Tafel linear regions at low overpotential and high overpotential. In the low over potential region, the estimated Tafel slopes are 69, 72, 84 and 90 mV/dec for Pt/C, BG-AgNW, RG-AgNW and BG respectively, confirming the first electron transfer as a primary rate-determining step. In the high over potential region, the Tafel slopes are 111, 119, 127 and 134 mV/dec for Pt/C, BG-AgNW, RG-AgNW and BG respectively. The variation from 69 mV to 120 mV in the catalysts may characteristically be ascribed to a transformation from Temkin to Langmuir conditions in the adsorption of oxygen intermediates; the transfer of the first electron to O_2_ is the rate-determining step in low and high current regions^62^,^63^,^64^. A close agreement between Tafel slopes of BG-AgNW and Pt/C also confirm the ORR pathway and rate determining step follow a similar path in both the catalysts. The plot of the potential verses electron transfer number (n) for AgNWs and BG-AgNWs are shown in Supplementary Fig. S16 at potential range from −0.3 V to −0.8 V. Further, the electron transfer number per O_2_ of the BG-AgNW was found to be ~4 in all the potential range, which is comparable to that of commercial Pt/C, confirming a direct 4 electron reduction pathway confirms the facile reaction kinetics on BG-AgNWs. The average ‘n’ values observed for BG, AgNW, RG-AgNWs and Pt/C are 3, 3.8, 3.4 and 3.9 respectively. A comparative study of current densities at –0.6 V is shown in Supplementary Table S1 (the performance of BG-AgNWs is greater than other reported silver and graphene-based materials in terms of current density, over potential difference and electron transfer number is obvious from this table). Rotating ring-disk electrode (RRDE) measurements were carried out to monitor the amount of hydrogen peroxide (H_2_O_2_) formed during the ORR process. Figure S17 displays the percentage of peroxide detected using BG-AgNWs electrode at 1600 rpm between –0.3 to –0.8 V. Remarkably, the measured H_2_O_2_ yield of BG-AgNWs was below 2% over the potential range of −0.3 to −0.8 V (versus Ag/AgCl), which was comparable to commercial Pt/C. The lower ring-current value for BG-AgNWs implies that very less amount of HO_2_^−^ reaches the Pt-ring electrode. The above results also confirm the four electron transfer process and the formation of lower percentage of H_2_O_2_ clearly demonstrated the high electrocatalytic ORR activity of BG-AgNWs. To evaluate the methanol tolerance of BG-AgNWs to methanol fuel, cyclic voltammetry in the O_2_ saturated 0.1 M KOH solution containing 1.0 M methanol and no noticeable change is observed for BG-AgNWs hybrid electrode towards ORR reaction (Fig. 6a). These results show that BG-AgNWs possess high selectivity for ORR with great tolerance against methanol, which is critical for applications in the direct alkaline fuel cells^41^. In addition BG-AgNWs exhibits excellent stability over a period of 25000 s at −0.4 V than Pt/C (Fig. 6b). Overall, the excellent ORR activity, high current density, good onset potential, better stability and superior tolerance towards methanol made BG-AgNWs as promising inexpensive cathodic electrocatalysts for alkaline fuel cells.

## Conclusions

In this work, we reported a strategy for the *in situ* formation of AgNWs over boron doped graphene sheets. Further, the crystal structure, surface chemical states of the synthesized BG-AgNWs was analysed using various spectroscopic and microscopy techniques. Results confirmed that the hybrid material will be an excellent candidate for fuel cell applications due to its excellent electrocatalytic activity and very high efficiency. The hybrid material follows an ideal four electron reduction pathway (n = 4), high current density (5.88 mAcm^−2^ at −0.6 V), high methanol tolerance, high stability and low yield of H_2_O_2_ (<2% at −0.8 V), which makes the BG electrode on par with Pt/C electrode. This feasible strategy presents a great capability of BG-AgNWs with wide applications in alkaline fuel cells and other various electrochemical energy devices.

## Methods

### Materials

Graphite powder, poly vinyl pyrrolidone (PVP, Mw ~55,000), silver nitrate (AgNO_3_) and boron oxide (B_2_O_3_) were purchased from Sigma- Aldrich. Analytical grade ethylene glycol (EG), potassium bromide (KBr), sodium chloride (NaCl) was purchased from Merck, India. All the solutions were prepared using Millipore water (Milli Q system).

### Synthesis of Boron doped graphene (BG)

The synthesis of Graphite oxide (GO) from graphite powder followed the modified Hummer’s method^65^,^66^. A detailed description followed for the synthesis of boron doped graphene (BG) was described in the previous work^67^,^68^. Briefly, GO and boron oxide (B_2_O_3_) were mixed in the 1:5 ratio and the entire mixture was kept in a quartz tube furnace and heated to 900 °C slowly at an increasing rate of 5 °C min^−1^ with a continuous flow of argon gas to facilitate an inert atmosphere in the tube furnace. In order to complete the doping process, the resulted product was kept at 900 °C for 3 hours, which was then cooled down to room temperature slowly under Ar atmosphere. The doped product BG was then treated with 3.0 M NaOH for 2 h in order to remove un-reacted boron oxide. After several washing steps and filtration, the BG was dried in vacuum at 60 °C. For a comparative study, reduced graphene (RG) was also synthesized using a similar procedure but without the addition of B_2_O_3_.

### Synthesis of Boron doped graphene silver nanowire (BG-AgNW)

10.0 mg of BG was dispersed in 20.0 mL of ethylene glycol (EG) and sonicated for 1 h. To this, 0.668 g of PVP was added. This mixture was then heated to 170 °C and then 0.01 g of KBr, 0.02 g of NaCl and finally 0.2793 g of AgNO_3_ were all added. The final solution was kept at 170° C for 6 hr to enhance the growth of AgNWs and allowed to cool down to room temperature (without using ice bath or other coolents). The product was then centrifuged and washed repeatedly with distilled water to remove any un-reacted chemicals. For comparison, RG-AgNWs was prepared by the same procedure by using RG instead of BG. The AgNWs was also synthesised by a similar protocol without the addition of BG.

### Material Characterization

XRD of the hybrid samples were performed using Bruker X-ray powder diffractometer with Cu-K α radiation of wavelength 1.541 Å. A confocal microprobe Raman system with an excitation wavelength of 532 nm was employed to obtain the Raman spectra of the samples. TEM analysis and selected area electron diffraction patterns (SAED) were performed using a JEOL JEM 2100 transmission electron microscope at an accelerating voltage of 200 kV. TEM samples were prepared by drop casting the sample over a carbon coated copper grid. Field emission scanning electron microscopy (FESEM) images with EDX mapping of the samples were performed by a field emission SEM system (FEI Quanta 400 ESEM FEG). For performing FESEM and EDX analysis, the samples were prepared by drop casting it on a silicon wafer. XPS analysis was carried out using PHI 5000 Versa Probe ULVAC instrument.

### Electrochemical Experiments

The electrochemical tests were measured on a Bio-Logic work station in a 0.1 M KOH solution in room temperature. Electrochemical performances were carried out using a three-electrode electrochemical cell. A glassy carbon electrode was used as a support electrode for the materials in the cyclic voltammetry measurements. An Ag/AgCl electrode in 3 M KCl aqueous solution was used as the reference electrode and a platinum wire as the counter electrodes, respectively. Glassy carbon electrode coated with the active material is attached to a rotating ring disk electrode was used as working electrode in the rotating ring electrochemical measurements. The catalysts coated electrode was prepared as follows: 100 μL of 5 wt% Nafion solution was added in 1 mL water/ethanol mixture, and then 4 mg of catalyst was dispersed in the as prepared solution and then sonicated for 60 min to form a homogeneous catalyst ink. Then 5 μL of the suspension was loaded onto a glassy carbon electrode of 3mm in diameter with a mass loading of about 0.283 mg cm^−2^. The catalyst inks were dried in air at room temperature. All catalysts were triggered by 20 cycles of cyclic voltammetry (CV) with a scan rate of 50 mV s^−1^ 0.2 to −0.8 V (vs Ag/AgCl) in N_2_ saturated 0.1 M KOH electrolyte solution before experiments. The linear sweep voltammetry measurements were conducted in the potential range from 0.2 V to −0.8 V at a scan rate of 10 mV s^−1^ versus Ag/AgCl in 0.1 M KOH. In the ORR experiment, the electrolyte was purged with high-purity O_2_ for 30 min before each test and maintained constant O_2_ gas flow during the measurements. Long-term stability test of BG was conducted by using the same set up with continuous O_2_ bubbling. All the experiments were conducted at room temperature (25 °C).

## Additional Information

**How to cite this article**: Nair, A. K. *et al*. Boron doped graphene wrapped silver nanowires as an efficient electrocatalyst for molecular oxygen reduction. *Sci. Rep.* **6**, 37731; doi: 10.1038/srep37731 (2016).

**Publisher's note:** Springer Nature remains neutral with regard to jurisdictional claims in published maps and institutional affiliations.

## Supplementary Material

Supplementary Information
