# Supplementary material for: Boron doped graphene wrapped silver nanowires as an efficient electrocatalyst for molecular oxygen reduction
Source: Sci Rep. 2016 Dec 12;6:37731. doi: 10.1038/srep37731 (PMC5150258; doi:10.1038/srep37731)
Supplement: Supplementary Information [file srep37731-s1.doc]

Supplementary Material

**Boron Doped Graphene Wrapped Silver Nanowires as an Efficient Electrocatalyst for Molecular Oxygen Reduction**

Anju K Nair,^a,e^ Vineesh Thazheveettil,^b^ Nandakumar Kalarikkal,^a,c*^ Sabu Thomas,^a,d^ Kala M S,^e^ Veena Sahajwalla,^f^ Rakesh K Joshi^f,*^ and Subbiah Alwarappan^b*^

^1^International and Inter University Centre for Nanoscience and Nanotechnology, Mahatma Gandhi University, Kottayam-686 560, Kerala, India

^2^CSIR- Central Electrochemical Research Institute (CSIR-CECRI) Karaikudi – 630 006, India.

^3^School of Pure and Applied Physics, Mahatma Gandhi University, Kottayam-686 560, Kerala, India

^4^School of Chemical Sciences, Mahatma Gandhi University, Kottayam-686 560, Kerala, India

^5^ Department of Physics,St Teresas’s College Ernakulam-682011, Kerala, India

^6^ The University of New South Wales, NSW, Australia

e.mail: [salwarap@gmail.com](mailto:salwarap@gmail.com) (SA); [nkkalarikkal@mgu.ac.in](mailto:nkkalarikkal@mgu.ac.in) (NK); [r.joshi@unsw.edu.au](mailto:r.joshi@unsw.edu.au) (RKJ)

**Synthesis of Silver Nanospheres (AgNS):**

The silver nanospheres were prepared by a polyol method^1^. Typically, 444 mg of PVP was added to 40 mL of PEG with stirring at 80 °C. When the solution was transparent, 1 mL of 0.5 M AgNO_3_ was added. This mixture was then transferred to an 80 mL Teflon-sealed autoclave and heated at 260 °C for 24 h. At the completion of the reaction, the solution was cooled to room temperature. The as prepared silver nanospheres were purified by centrifugation at 16,000 rpm for 20 min. The product was then dispersed in DI water or ethanol and centrifuged to remove the PEG and PVP.

**Synthesis of Ag Nanocubes (AgNC):**

In a typical synthesis^2^, 5 mL of ethylene glycol (EG) was added into a 100 mL round-bottomed flask and heated under magnetic stirring in an oil bath preset to 150 °C followed by the addition of Sodium hydrosulphide (NaHS) (0.06 mL, 3 mM in E G). To this, HCl (0.5 mL, 3 mM in EG) was added followed by the addition of poly (vinyl pyrrolidone) (PVP-55, MW ≈ 55 000, 1.25 mL, 20 mg/mL in EG). To this mixture, silver trifluoroacetate (CF_3_COOAg) (0.4 mL, 282 mM in EG) was then added into the mixture. During the entire procedure, the flask was capped with a glass stopper except while adding reagents. The Ag nanocubes were obtained by quenching the reaction with an ice-water bath.

**RDE measurements:**

Linear Sweep voltammetry (LSV) was performed at RDE loaded with different samples at different rotation rates to investigate the electrode kinetics towards ORR. The overall electron transfer number (n) per oxygen molecule in a typical ORR process at different electrodes can be calculated by using Koutechy-Levich (K-L) equation as follows

1/ i = 1/i_k_ +i/i_L_ = (nFAkC0)^-1^ + 0.2nFAD^2/3^C_0_v ^-1/6^ ω^1/2^)^-1^

where I is the measured current, i_k_ and i_L_ are the kinetic and diffusion–limiting current respectively, n is the electron transfer number per oxygen molecule, F is the Faraday constant, A is the geometrical area of the electrode; D is the diffusion coefficient of O_2_ in 0.1 M NaOH. C_0_ is the bulk concentration of O_2_ (1.2 x 10^-3^ mol/ litre), new is the kinematic viscosity of electrolyte (0.01 cm^2^/s), ω is the rotation in rpm and k is the electron transfer rate constant. From the corresponding K-L plots (i^-1^ vs. ω ^-1/2^), it can be seen that the data exhibited good linearity.

The n can be calculated from the reciprocal of the slope (B), ie

B= 0.2nFAD^2/3^C_o_v^-1/6^

**RRDE Experiment:**

The four-electron selectivity of the catalyst was evaluated based on the H_2_O_2_ yield. The disc electrode undergoing cathodically scanned at a scan rate of 10mV/s in and the ring potential (0.5 V vs Ag/AgCl) was constant for oxidizing any HO_2_^-^ intermediate.

The H_2_O_2_ yield was determined by the following equations:


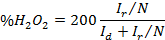


where, I_D_ is the disk current, and I_R_ is the ring current, and N is current collection efficiency

of the Pt ring. N is 0.41 from the reduction of K3 [Fe (CN)_6_]. From RRDE, the electron transfer number (n) can also be calculated by the equation


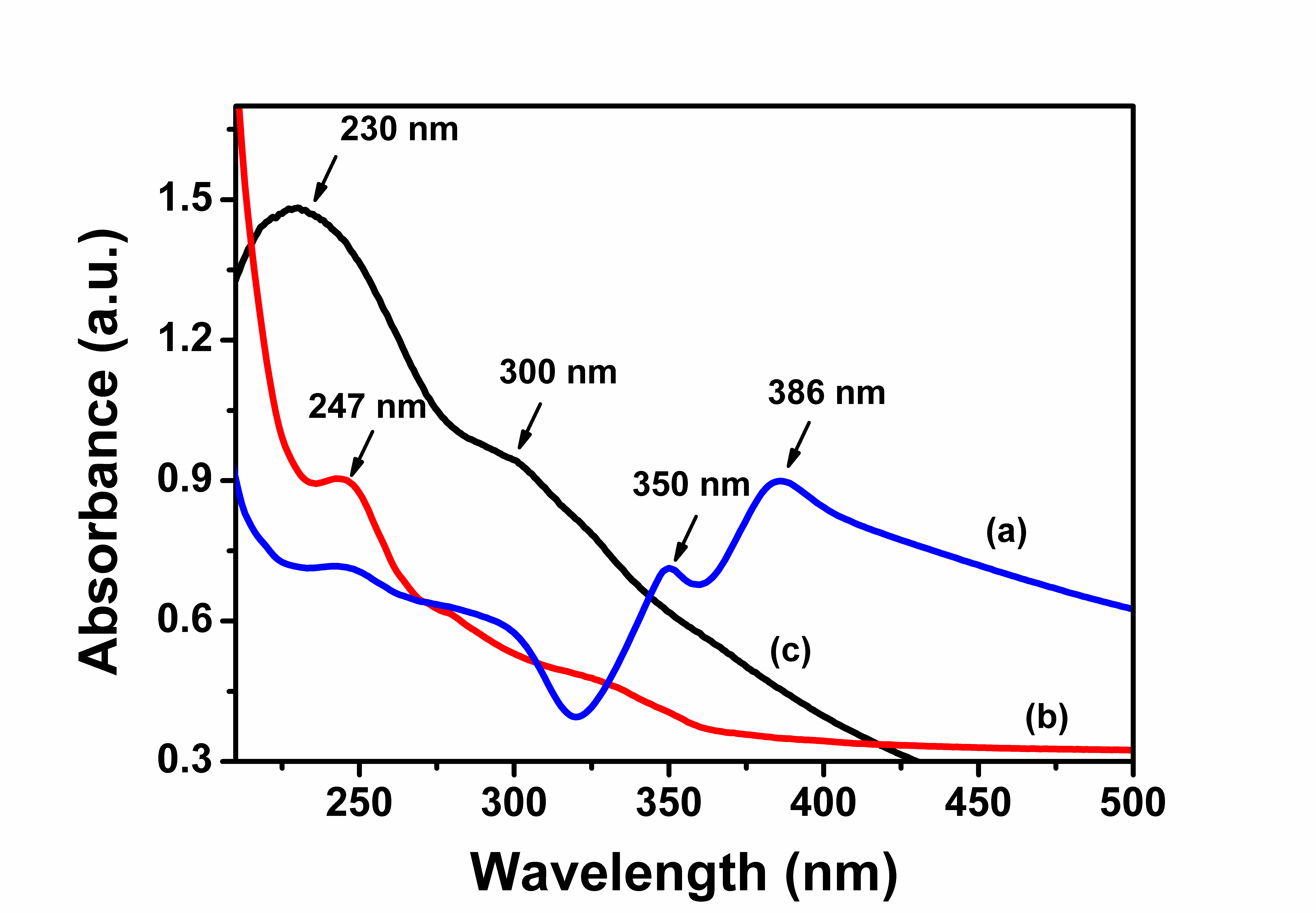
n=4 x (I_D_/ (I_D_+I_R_/N))

**Figure S1.** UV-Vis absorption spectra of (a) BG-AgNW (b) BG and (c) GO

**
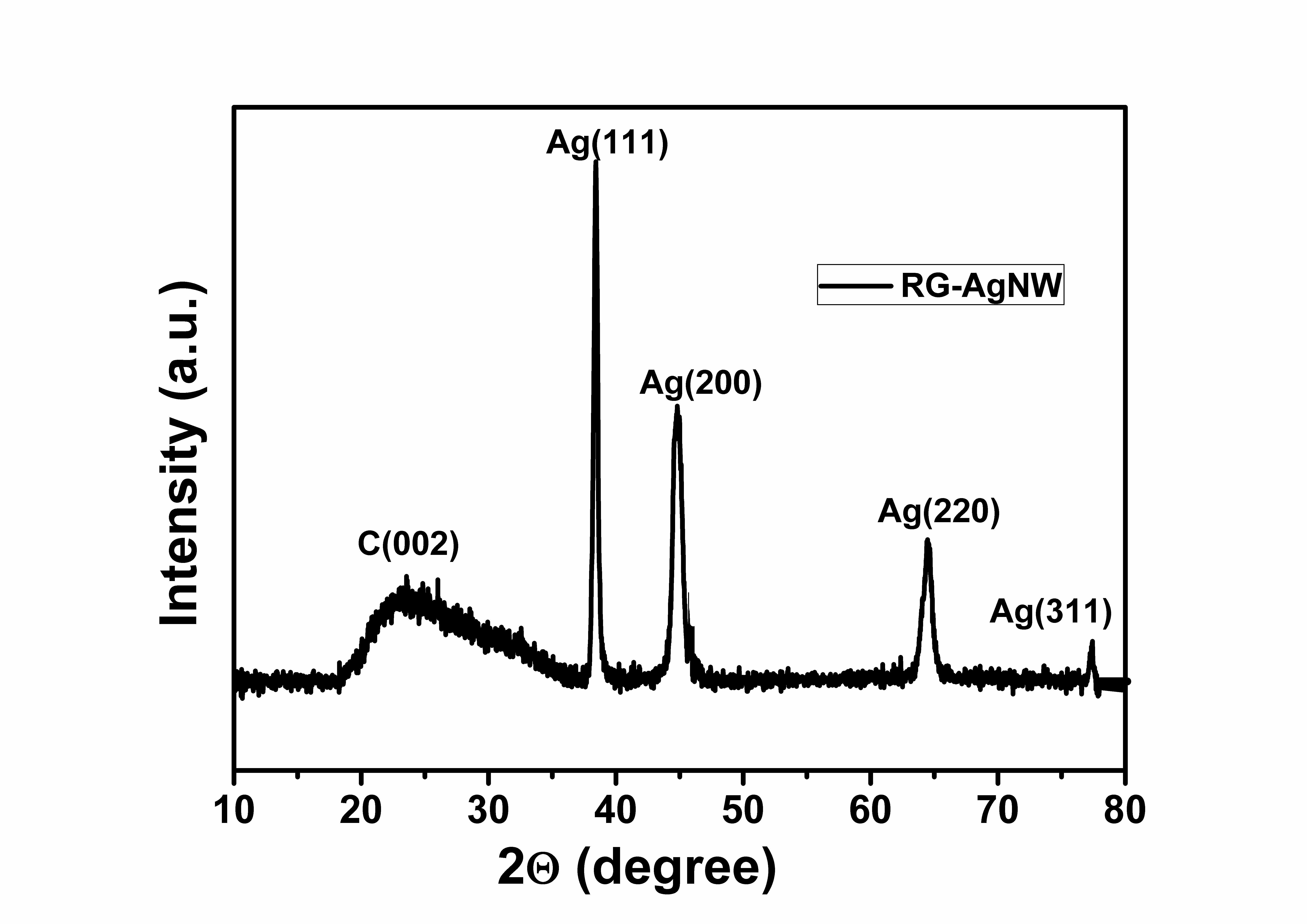
**

**Figure S2.** XRD spectrum of RG-AgNW


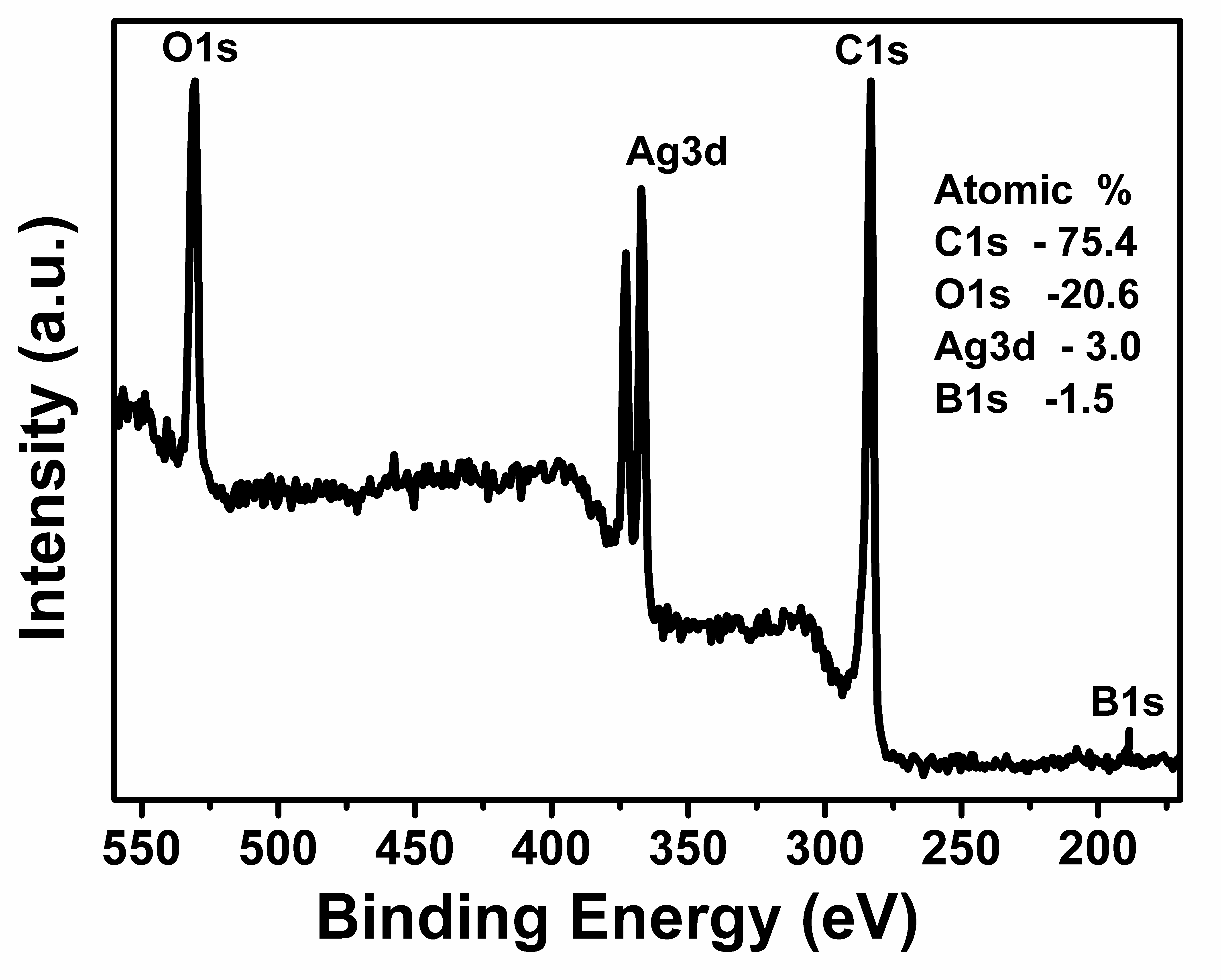


**Figure S3.** XPS survey spectrum of BG-AgNW


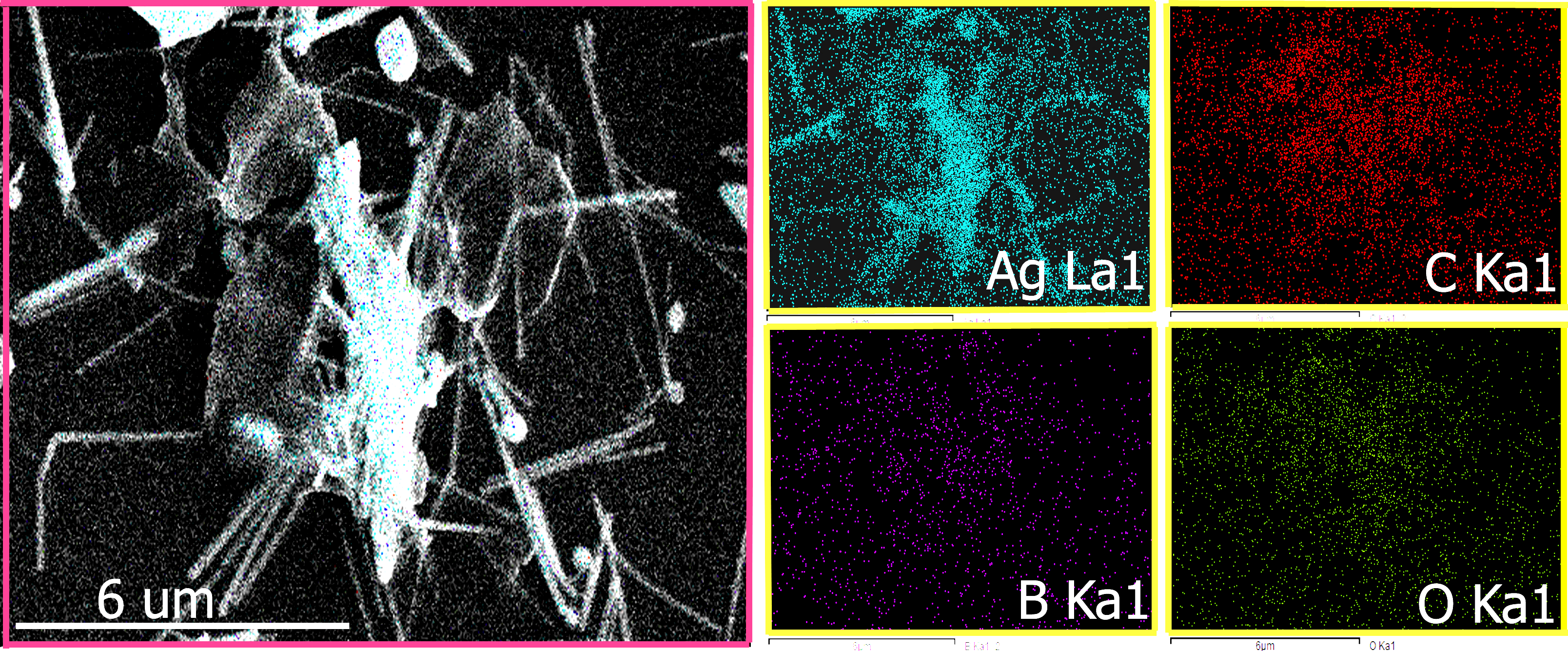


**Figure S4.** EDX elemental mapping of BG-AgNW


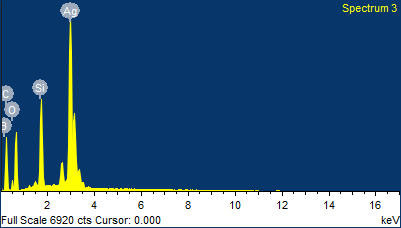


**Figure S5.** EDX spectrum of BG-AgNW

**
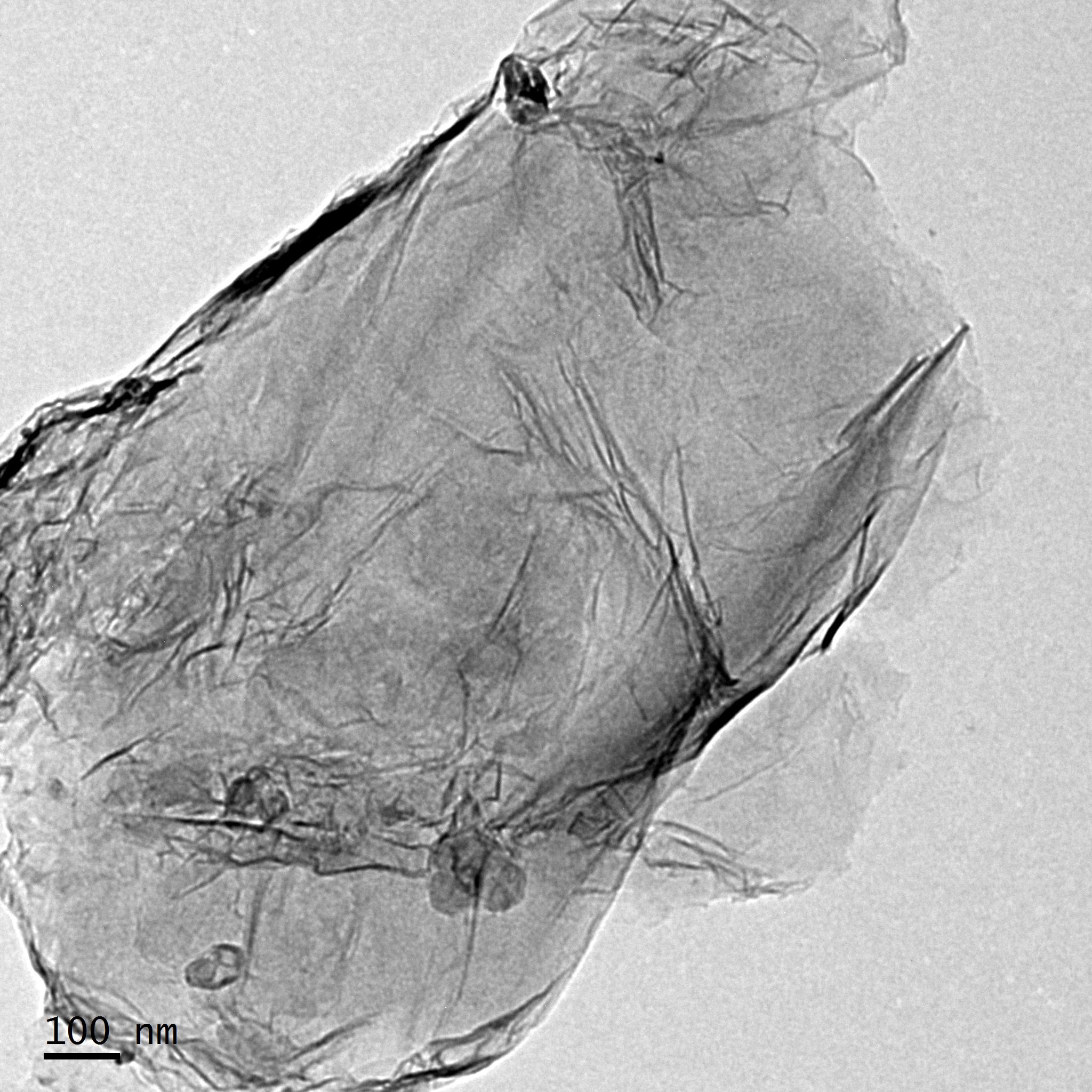
**

**Figure S6**. TEM image of BG

**
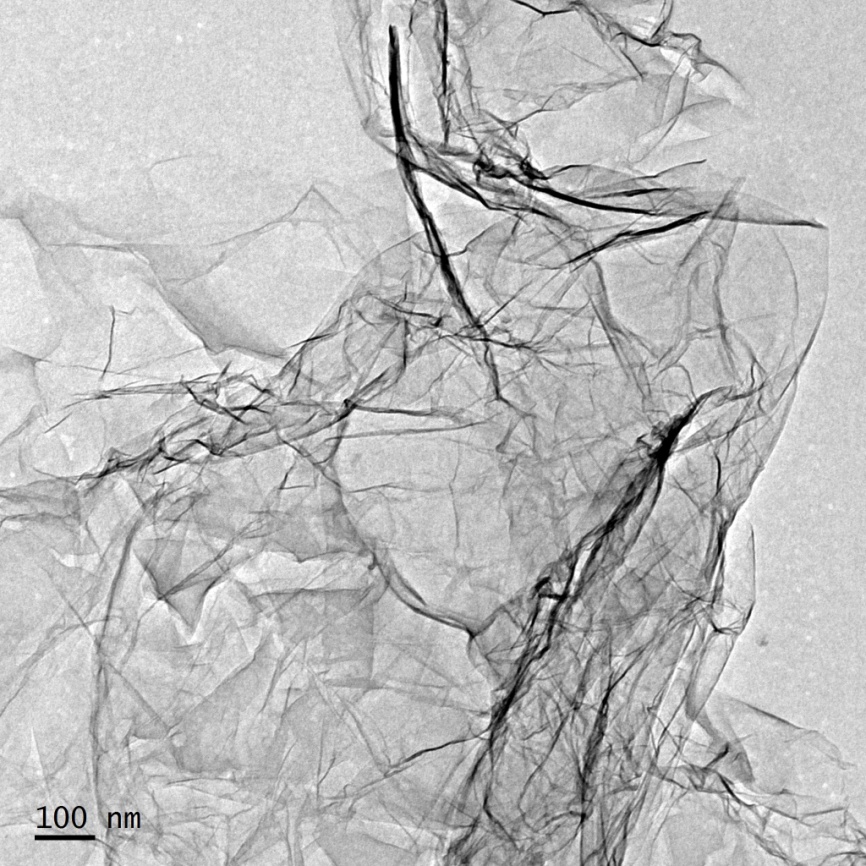
**

**Figure S7.** TEM image of RG

**
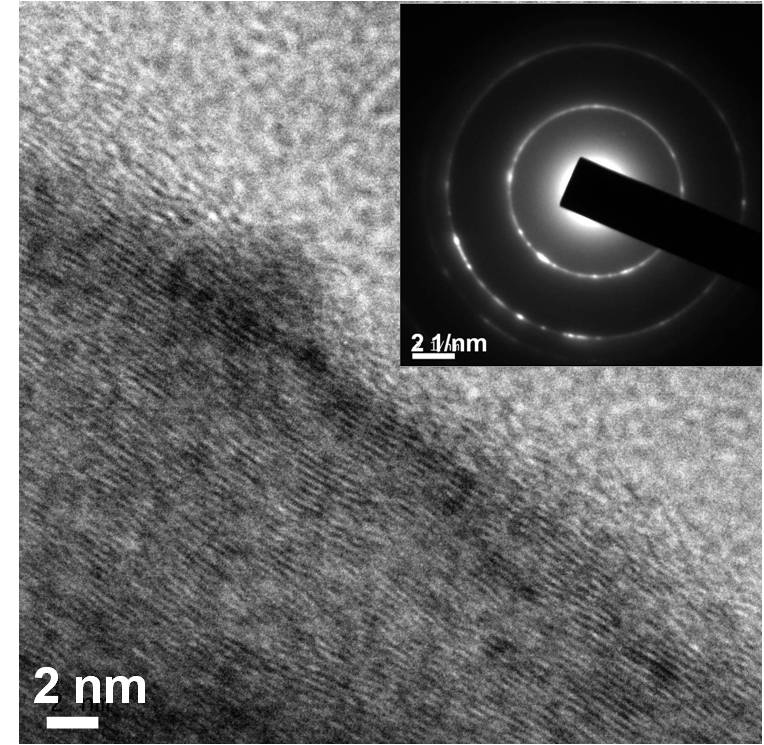
**

**Figure S8.** HRTEM image of BG, The inset shows the corresponding SAED pattern


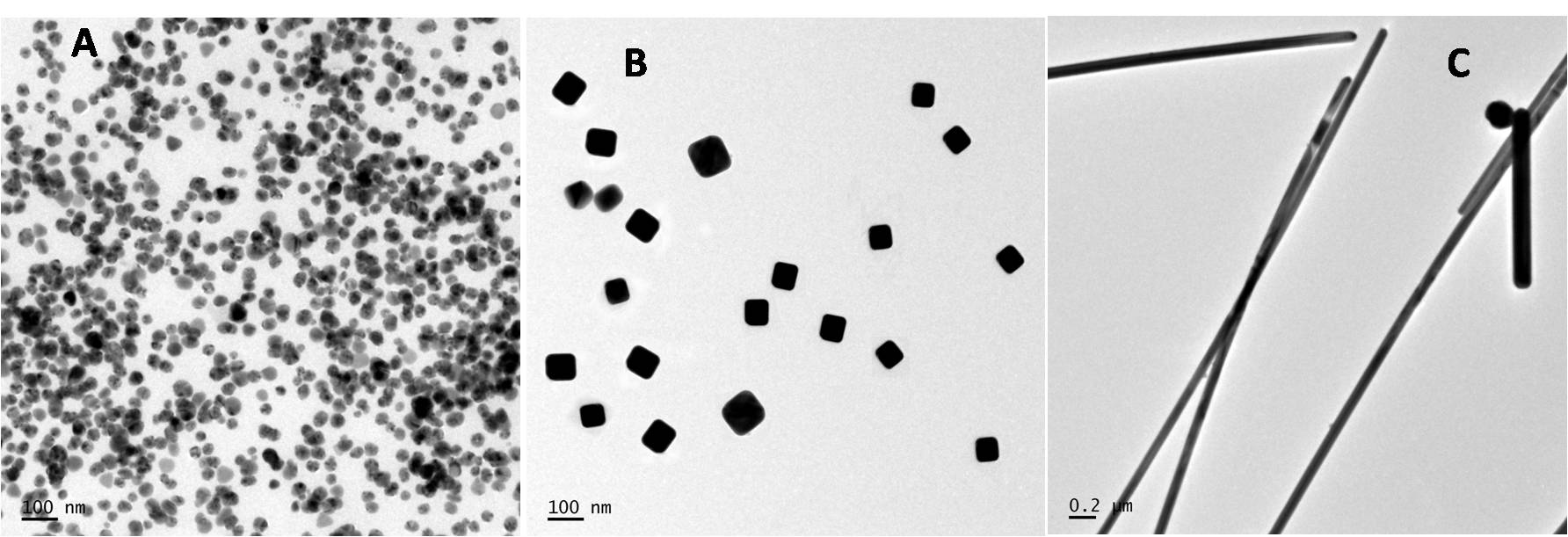


**Figure S9.** TEM images of (A) AgNS (B) AgNC and (C) AgNW

**
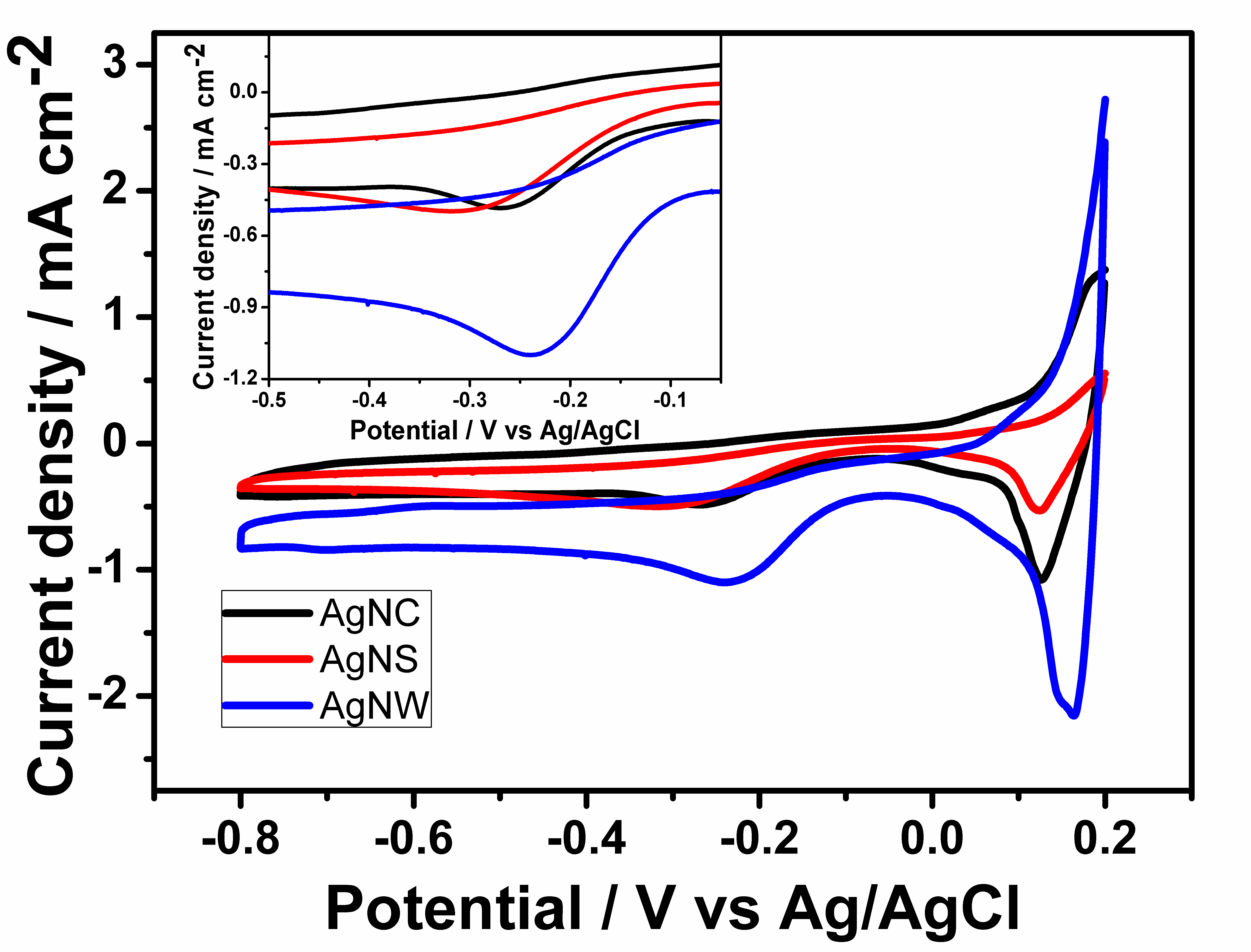
**

**Figure S10.** CVs of AgNC, AgNS and AgNW in O_2_ saturated 0.1 M KOH


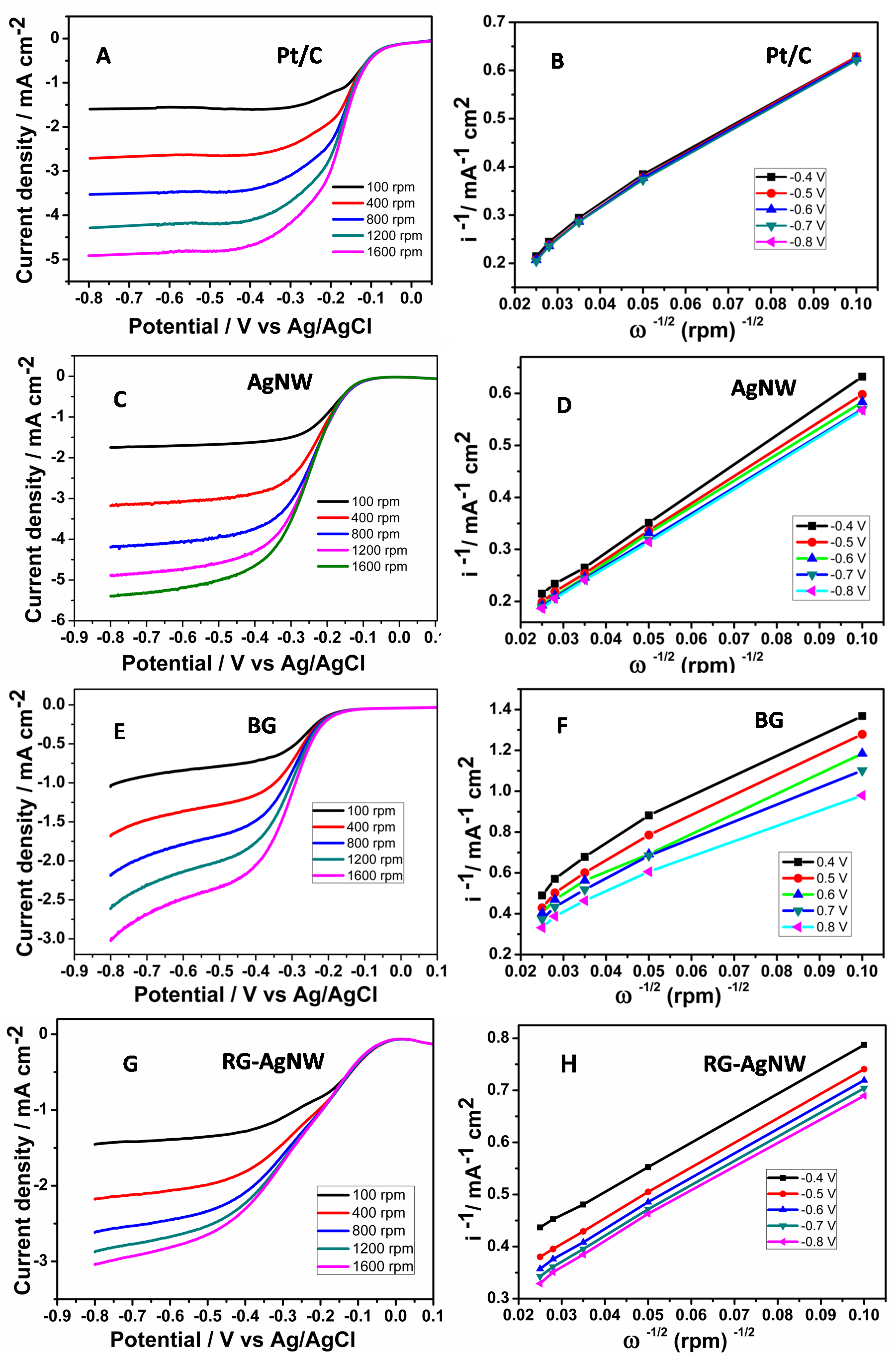


**Figure S11**. RDE experiments for (A) Pt/C (C) AgNW (E) BG and (G) RG-AgNW respectively and corresponding K-L plots for (B) Pt/C (D) AgNW (F) BG (H) RG-AgNW


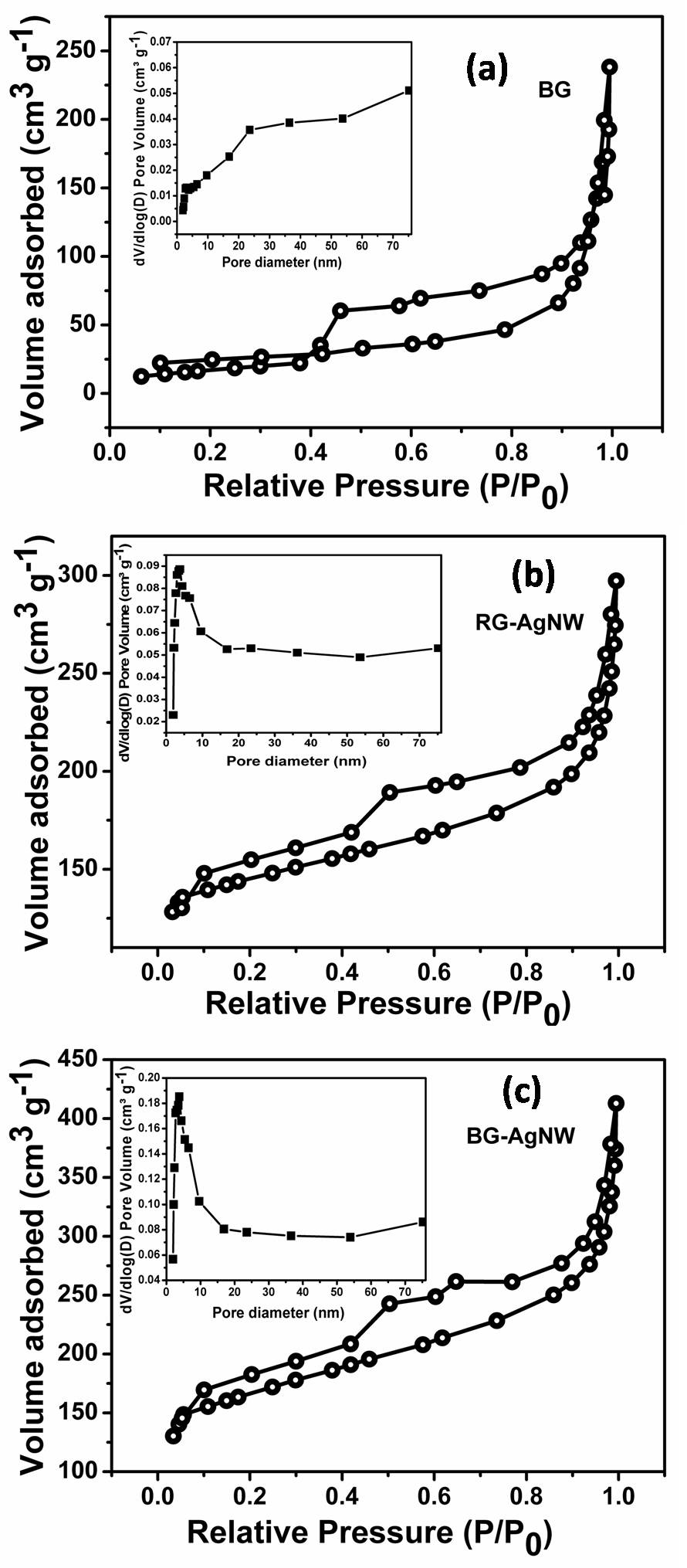


**Figure S12.** Nitrogen adsorption-desorption isotherm of (a) BG (b) RG-AgNW and (c) BG-AgNW; the inset shows the corresponding pore size distribution curve of BG, RG-AgNW and BG-AgNW respectively.


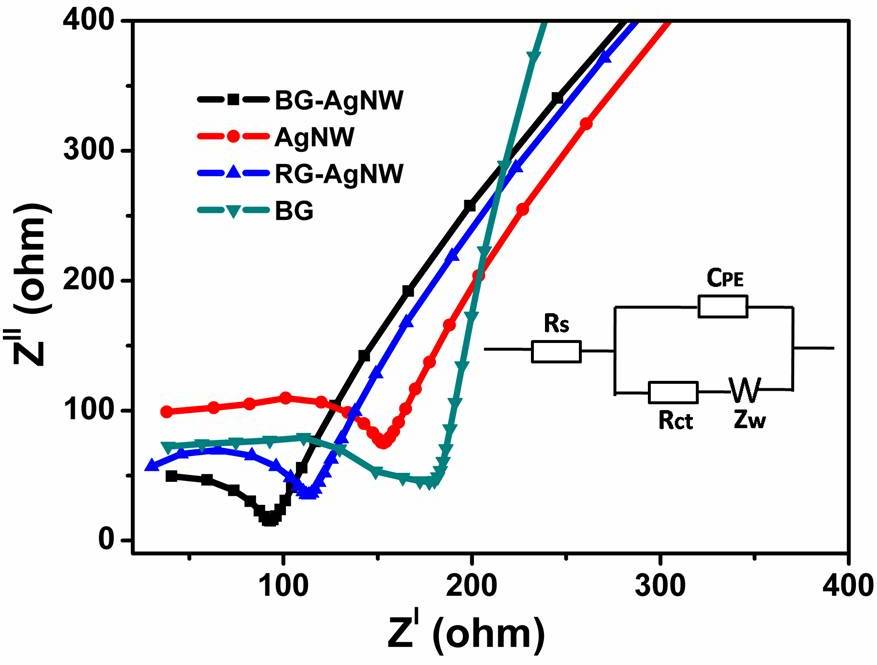


**Figure S13.** The electrochemical impedence spectra of BG, AgNW, RG-AgNW and BG-AgNW: the inset shows the equivalent Randles circuit, Rs corresponds to the solutions resistance, C_PE_ constant phase element, and Rct is the charge transfer resistance


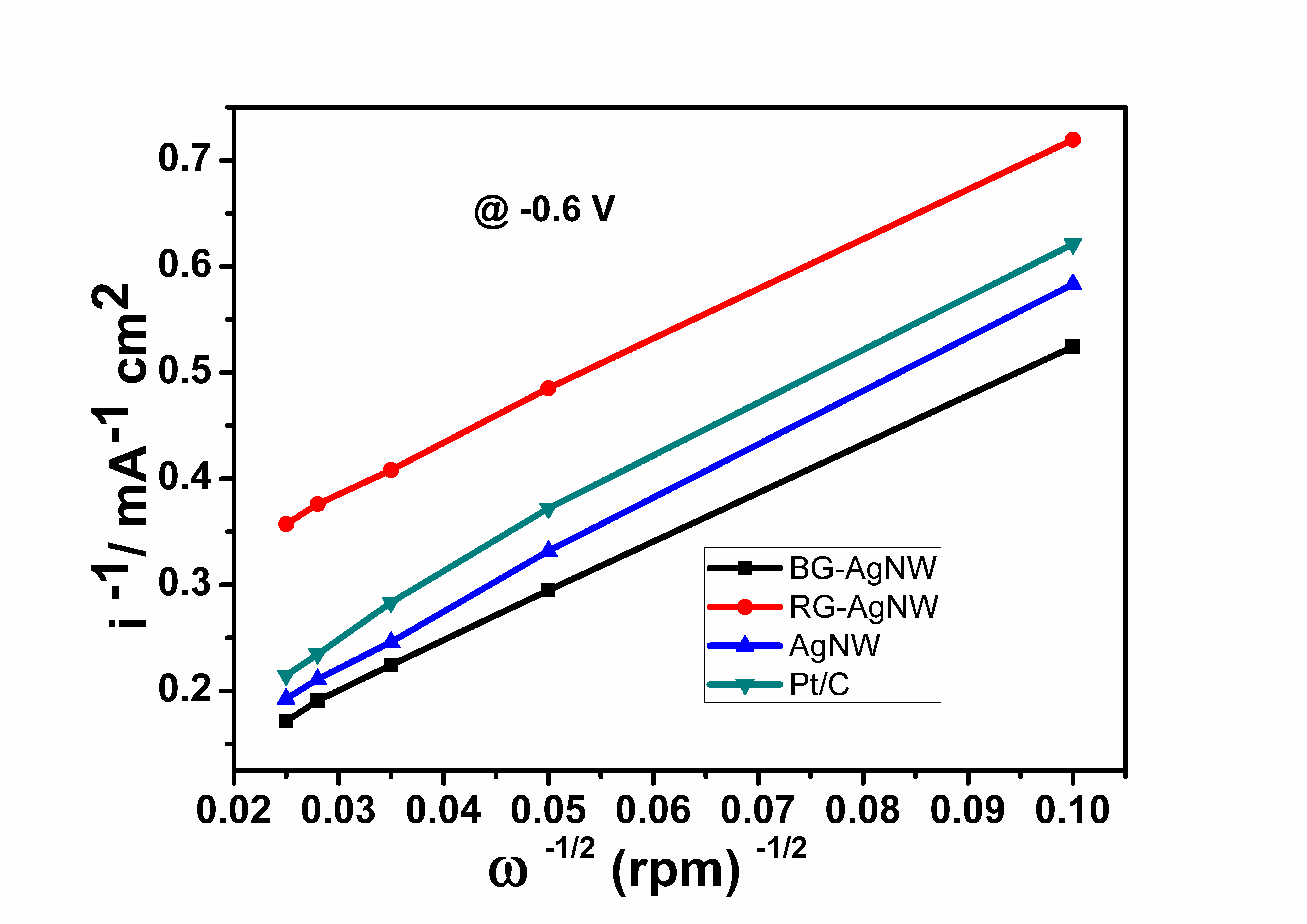


**Figure S14.** Comparison of K-L plots at -0.6 V


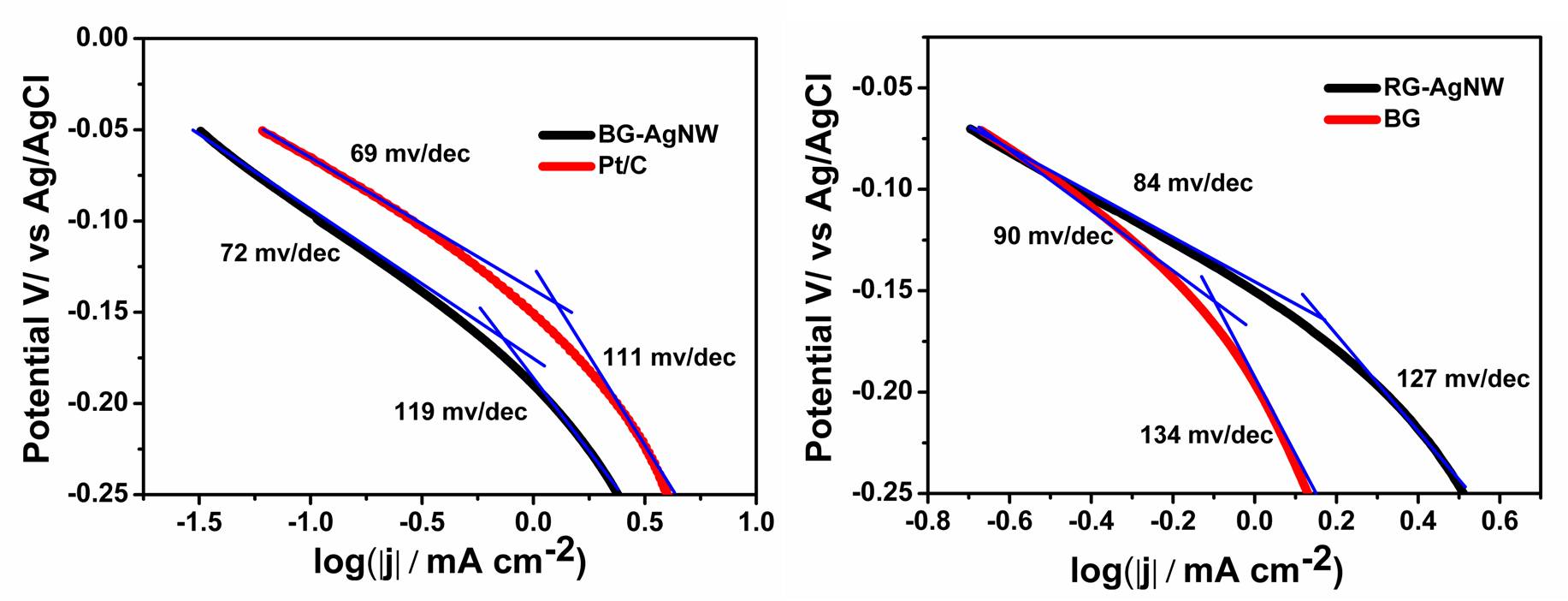


**Figure S15.** Tafel plots for Pt/C, BG-AgNW, RG-AgNW and BG


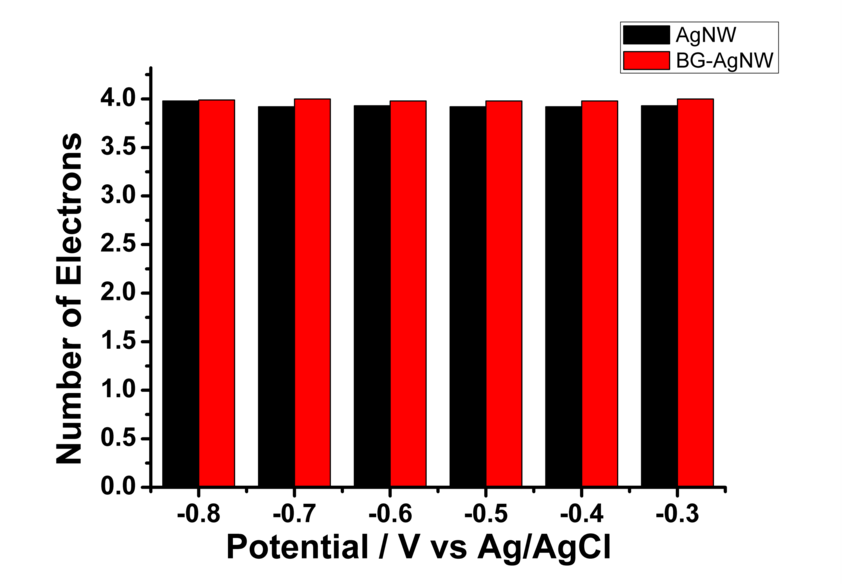


**Figure S16.** Plot of Potential against number of electron


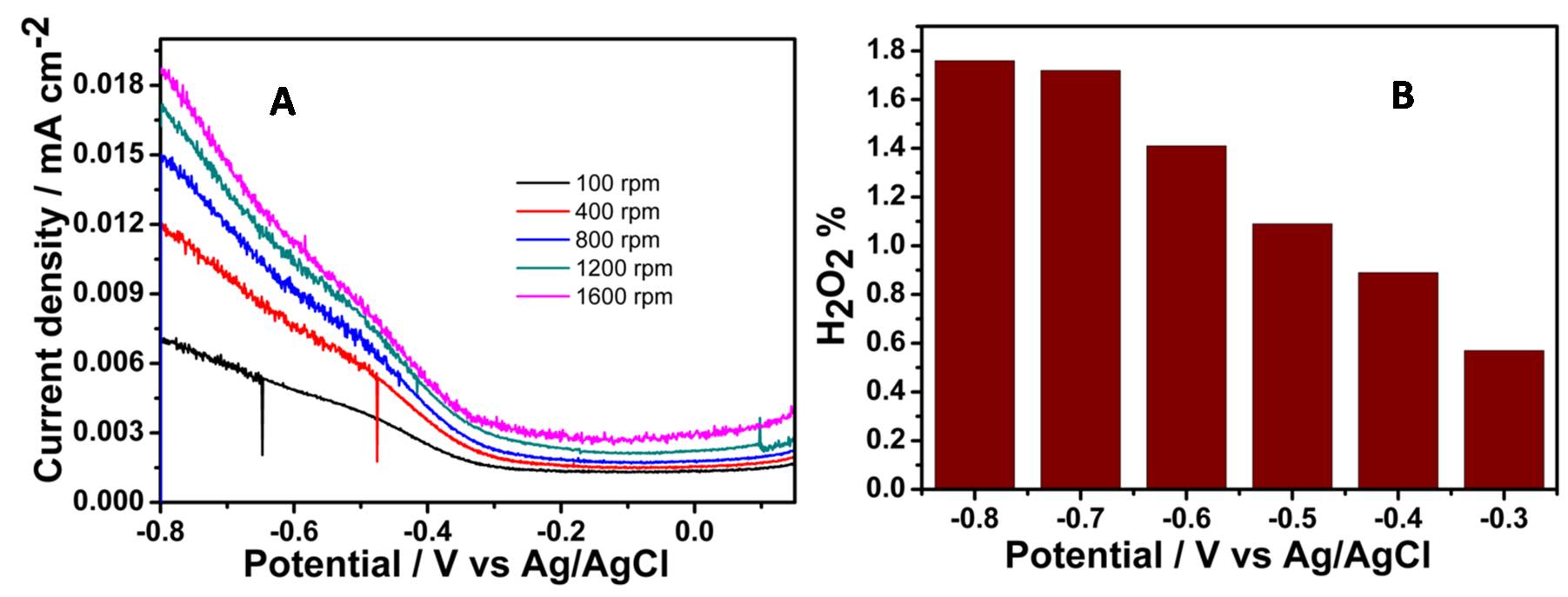


**Figure S17.** Ring current taken from BG-AgNW coated on RRDE electrode at different rpm (b) Corresponding plot for percentage of peroxide formed at different potentials.

**Table S1.** ORR performances of various types of silver and graphene samples

| Items | Catalyst loading (mg cm^-2^) | Electron transfer number (n) | Current density @ -0.6V  (mA cm^-2^) | Over potential difference w.r.t Pt/C (mV) | References |
| --- | --- | --- | --- | --- | --- |
| BG-AgNW | 0.283 | 4 | 5.88 | 20 (*Ref*. Ag/AgCl) | This work |
| AgNW | 0.283 | 3.9 | 5.1 | 65 (*Ref*. Ag/AgCl) | This work |
| BG | 0.283 | 3 | 2.5 | 160 (*Ref*. Ag/AgCl) | This work |
| B-nanotube | 0.255 | 2.5 |  | 150 (*Ref.* SCE) | [3] |
| Ag small triangle plate | - | 3.27 | 2.24 | - | [4] |
| BG | 0.283 | 3.9 | 4.65 | 59 (*Ref*. Ag/AgCl) | [5] |
| N-graphene | 0.255 | 3.3 | 3.10 | 120 (*Ref*.Ag/AgCl) | [6] |
| Ag/C | - | 3.09 |  | 236 (*Ref*. Ag/AgCl) | [7] |
| Ag/GNR | - | 3.51 |  | 82 (*Ref*. Ag/AgCl) | [7] |
| B-graphene | 0.283 | 3.09 | 1.90 | - | [8] |
| N-graphene | 0.283 | 2.91 | 2.33 | - | [8] |
| B,N-graphene | 0.283 | 3.81 | 5.01 | - | [8] |
| h-BN/graphene | 0.283 | 3.28 | 2.42 | - | [8] |

**References**

1. Hongyan, L. *et.al.* Controlled Synthesis of Uniform Silver Nanospheres. *J. Phys. Chem. C,* **114,** 7427-7431(2010)

2. Qiang , Z. *et.al.* Facile Synthesis of Ag Nanocubes of 30 to 70 nm in Edge Length with CF_3_COOAg as a Precursor. *Chem. Eur. J.* **16**, 10234 – 10239 (2010).

3. Yang, L. *et.al.* Boron doped carbon nanotubes as metal free electrocatalysts for the oxygen reduction reaction. *Angew. Chem. Int. E.* **50**, 7132-7135 (2011).

4. Lee, C. L., Chiou, H. P., Syu, C. M. & Wu, C. C. Silver triangular nanoplates as electrocatalyst for oxygen reduction reaction. *Electrochemistry Communications*. **12**, 1609-1613 (2010).

5. Vineesh, T. V. *et.al*. Bifunctional electrocatalytic activity of boron-doped graphene derived from boron carbide. *Adv. Energy Mater.* ***1500658***,1 (2015).

6. Lin, Z., Song, M., Ding, Y., Liu, Y., Liua, M. & Wong, C. Facile preparation of nitrogen-doped graphene as a metal-free catalyst for oxygen reduction reaction. *Phys. Chem.Chem. Phy.* **14**, 3381-3387 (2012).

7. Davis, D. J. *et.al.* Silver-graphene nanoribbon composite catalyst for the oxygen reduction reaction in the alkaline electrolyte. *Electroanalysis.* **26**, 164-170 (2014).

8. Zheng, Y., Jiao, Y., Ge, L., Jaroniec, M. & Qiao, S. Z. Two step boron and nitrogen doping in graphene for enhanced synergistic catalysis. *Angew. Chem. Int. Ed,* **52,** 3110-3116 (2013).
